# Analysis of the Genetic Diversity and Population Structure of Austrian and Belgian Wheat Germplasm within a Regional Context Based on DArT Markers

**DOI:** 10.3390/genes9010047

**Published:** 2018-01-22

**Authors:** Mohamed A. El-Esawi, Jacques Witczak, Abd El-Fatah Abomohra, Hayssam M. Ali, Mohamed S. Elshikh, Margaret Ahmad

**Affiliations:** 1 UMR CNRS 8256 (B2A), Université Paris VI, 75005 Paris, France; jacques.witczak@upmc.fr (J.W.); margaret.ahmad@upmc.fr (M.A.); 2 Botany Department, Faculty of Science, Tanta University, Tanta 31527, Egypt; 3 School of Energy and Power Engineering, Jiangsu University, Zhenjiang 212013, China; abomohra@yahoo.com; 4 Botany and Microbiology Department, College of Science, King Saud University, Riyadh 11451, Saudi Arabia; hayhassan@ksu.edu.sa (H.M.A.); melshikh@ksu.edu.sa (M.S.E.); 5 Timber Trees Research Department, Sabahia Horticulture Research Station, Horticulture Research Institute, Agriculture Research Center, Alexandria 21526, Egypt; 6 Department of Biology, Xavier University, Cincinnati, OH 45207, USA

**Keywords:** wheat, genetic diversity, population structure, relationships, diversity array technology markers

## Abstract

Analysis of crop genetic diversity and structure provides valuable information needed to broaden the narrow genetic base as well as to enhance the breeding and conservation strategies of crops. In this study, 95 Austrian and Belgian wheat cultivars maintained at the Centre for Genetic Resources (CGN) in the Netherlands were characterised using 1052 diversity array technology (DArT) markers to evaluate their genetic diversity, relationships and population structure. The rarefacted allelic richness recorded in the Austrian and Belgian breeding pools (*A*_25_ = 1.396 and 1.341, respectively) indicated that the Austrian germplasm contained a higher genetic diversity than the Belgian pool. The expected heterozygosity (*H_E_*) values of the Austrian and Belgian pools were 0.411 and 0.375, respectively. Moreover, the values of the polymorphic information content (PIC) of the Austrian and Belgian pools were 0.337 and 0.298, respectively. Neighbour-joining tree divided each of the Austrian and Belgian germplasm pools into two genetically distinct groups. The structure analyses of the Austrian and Belgian pools were in a complete concordance with their neighbour-joining trees. Furthermore, the 95 cultivars were compared to 618 wheat genotypes from nine European countries based on a total of 141 common DArT markers in order to place the Austrian and Belgian wheat germplasm in a wider European context. The rarefacted allelic richness (*A*_10_) varied from 1.224 (Denmark) to 1.397 (Austria). Cluster and principal coordinates (PCoA) analyses divided the wheat genotypes of the nine European countries into two main clusters. The first cluster comprised the Northern and Western European wheat genotypes, whereas the second included the Central European cultivars. The structure analysis of the 618 European wheat genotypes was in a complete concordance with the results of cluster and PCoA analyses. Interestingly, a highly significant difference was recorded between regions (26.53%). In conclusion, this is the first study to reveal the high diversity levels and structure of the uncharacterised Austrian and Belgian wheat germplasm maintained at the CGN as well as place them in a wider European context. The results should help plant breeders to utilise the most promising wheat genotypes of this study in future breeding programmes for enhancing wheat cultivars.

## 1. Introduction

Wheat (*Triticum aestivum* L.) is an important allohexaploid cereal crop that originates from three diploid species: *Triticum urartu* (AA), a progenitor related to *Aegilops speltoides* (BB), and *Aegilops tauschii* (DD) [1]. Wheat is adaptable to grow in diverse environments, extending from the Caspian Sea to China [2,3]. Domestication has resulted in reducing the genetic variability in several crop species, including wheat [4]. The narrow genetic base is a major concern threatening crop genetic improvement progress [3]. A significant decrease in the genetic diversity of different crop cultivars registered during the last century has also been recorded [5]. Introgression of novel alleles from various plant genetic resources can enhance the reduced genetic diversity. Characterization and exploitation of the germplasm maintained at the plant genetic resource centres are, therefore, essential to enhance crop yield and resistance to pathogens in order to meet the needs of the growing human population [6]. Hundreds of thousands of wheat accessions, representing diverse geographical locations worldwide, have been maintained at numerous plant genetic resource centres without appropriate genetic characterization or revealing their potential value in crop improvement [1,7].

Characterisation of the genetic diversity and phylogenetic relationships provides valuable information needed to broaden the narrow genetic base as well as enhance breeding and conservation strategies for crops [8,9,10,11]. Analysis of population structure also facilitates a deeper understanding of germplasm diversity and association mapping studies [3,12]. Therefore, various approaches including coefficient of parentage, morphological traits and biochemical markers (storage proteins and isozymes) have been used for evaluating the genetic variation level and structure in many crops, including wheat, maize, soybeans and barley [13,14,15,16,17,18,19,20]. Analysis of genetic diversity based on these phenological, morphological and biochemical traits is affected by the environmental factors [3]. Therefore, molecular DNA markers have been developed and proved powerful in genetic diversity analysis.

DNA markers such as inter-simple sequence repeats (ISSR), amplified fragment length polymorphism (AFLP), restriction fragment length polymorphism (RFLP), microsatellites, and single nucleotide polymorphisms (SNPs) have been utilised to assess the relationships and genetic diversity levels in wheat germplasm [21,22,23,24,25,26]. Among these markers, microsatellites have been the most commonly suitable approaches in the plant genetic diversity analysis as they are abundant, codominant, highly polymorphic and widely distributed along chromosomes [22,27,28,29,30]. DNA markers have been also developed to involve diversity array technology (DArT [31]), which is a high-throughput microarray-based method used for genome profiling, association mapping, fingerprinting and assessing genetic diversity and structure in many crops, including wheat [32]. Diversity array technology markers have been further used and proved efficient for more wheat genetic diversity studies [1,3,33,34,35].

The main objectives of the current research were to utilise DArT markers to (i) evaluate the genetic diversity of Austrian and Belgian wheat breeding pools maintained, without appropriate genetic characterization, at the Centre for Genetic Resources (CGN) in the Netherlands; (ii) analyse the population structure of the Austrian and Belgian genetic pools, and reveal new information regarding their breeding and genetic structure; and (iii) place the Austrian and Belgian germplasm in a wider European context through accessing and exploiting a combined set of available DArT genotypes.

## 2. Materials and Methods

### 2.1. Plant Germplasm

#### 2.1.1. Austrian and Belgian Wheat Breeding Pools

A set of 70 Austrian and 25 Belgian accessions of wheat (*T. aestivum* L.), maintained at the CGN, was obtained and analysed in the present study (Table S1).

#### 2.1.2. European Wheat Breeding Pool

In order to place the Austrian and Belgian wheat breeding pools in a wider European context, DArTs accessible from three wheat pools previously published were involved in the current study, (i) The TriticeaeGenome panel comprising 376 wheat cultivars from the UK, Germany and France [35]; (ii) The European diversity panel comprising 94 European wheat cultivars [30]; and (iii) The Croatian wheat panel comprising 89 wheat cultivars [3]. The numbers of cultivars in each panel and represented countries are summarised in Table 1.

### 2.2. DNA Extraction and DArT Analysis

Genomic DNA (gDNA) was isolated from the young leaf tissue of each of the Austrian and Belgian wheat accessions using DNeasy Plant Mini Kit (Qiagen, Marseille, France) and was subjected to DArT analysis according to the general procedures previously described [31]. Out of all DArT markers scored, 1052 markers of a higher quality were selected for further genetic analysis of the Austrian and Belgian breeding pools. Furthermore, DArT data representing the European wheat breeding pool was accessed from the three published wheat panels [3,34,35]. Across the Austrian and Belgian wheat panels as well as the European wheat breeding pool, only countries of at least 10 varieties and common markers were included in the analysis. The final combined dataset of the Austrian and Belgian wheat panels and the European wheat breeding pool comprised 618 cultivars from nine countries and 141 common DArT markers (Table 1).

### 2.3. Data Analysis

#### 2.3.1. Genetic Diversity and Population Structure of Austrian and Belgian Wheat Pools

DArT-based diversity analysis of the Austrian and Belgian wheat cultivars was assessed using different diversity indices. Since the Austrian and Belgian breeding pools are different in sample size, rarefaction method was used to calculate the rarefacted allelic richness of the two pools, in a standardised sample size equivalent to the smallest population size of *n* = 25 (*A*_25_) using FSTAT version 2.9.3 [36]. The polymorphic loci percentage (%*P*), effective number of alleles (*N_E_*), expected heterozygosity (*H_E_*), and polymorphic information content (*PIC*) were also calculated using GenAlex v. 6.41 [37] and PowerMarker v. 3.25 [38].

A Jaccard dissimilarity matrix-based neighbour-joining tree with 1000 bootstraps was conducted for each of the Austrian and Belgian breeding pools, separately. The population structure analysis was conducted for each of the Austrian and Belgian breeding pools using Structure 2.4.1 software [39]. The hypothetical populations’ number (*k*) varied from 1 to 15, with 100,000 burn-in run iteration, followed by 100,000 Markov chain Monte Carlo replicates. Ten independent iterations were run. Structure Harvester [40] was then used to calculate the most likely number of *k* [41]. Analysis of molecular variance (AMOVA) was conducted to partition the molecular diversity between and within the populations (*k*), generated by Structure analysis, of each of the Austrian and Belgian wheat breeding pools using GenAlex v. 6.41 [37].

#### 2.3.2. Diversity, Population Structure and Relationships among Wheat Varieties from Nine Countries

Based on the 141 common DArT markers, the genetic diversity of the European wheat varieties from nine countries (Austria, Belgium, Croatia, Germany, France, Denmark, Sweden, Hungary and the UK) has been estimated using the same diversity indices mentioned above (%*P*, *H_E_*, *N_E_*, *PIC*). Rarefaction was used to calculate the rarefacted allelic richness of the nine European populations in a standardized sample size equivalent to the smallest population size of *n* = 10 (*A*_10_) using FSTAT version 2.9.3 [36].

Analysis of molecular variance analysis was performed to partition the molecular diversity between regions (Northern and Western Europe vs. Central Europe), within countries, and among countries within regions. The Northern and Western European region contained Belgium, Germany, Denmark, Sweden, France, and UK. The Central European countries included Austria, Croatia, and Hungary. The genetic differentiation (*F_ST_*) values were calculated between pairs of countries and were used to construct the cluster analysis among the nine countries using the Unweighted pair group method with arithmetic mean (UPGMA) method. To reveal the relationships among the 618 wheat accessions representing the nine European countries, principal coordinate analysis (PCoA) was conducted using GenAlEx 6.41 software [37]. The genetic structure of all European wheat cultivars from the nine countries was evaluated using Structure software ver. 2.4.1, as described above. 

## 3. Results

### 3.1. Genetic Diversity and Population Structure of Austrian and Belgian Wheat Pools

In this study, a total of 1052 DArT markers were used to assess the genetic diversity and structure of 95 Austrian and Belgian wheat cultivars. Table 2 shows the diversity indices estimated for the Austrian and Belgian pools based on 1052 DArT markers. The values of rarefacted allelic richness (*A*_25_) recorded in the Austrian and Belgian breeding pools were 1.396 and 1.341, respectively, indicating that the Austrian breeding pool contained a slightly higher diversity than the Belgian pool. Furthermore, the percentage of polymorphic markers recorded in the Austrian and Belgian breeding pools was 93.79% and 91.46%, respectively (Table 2). The average values of the effective number of alleles per locus (*N_E_*) found in the Austrian and Belgian breeding pools were 1.698 and 1.602, respectively. The *H_E_* values of the Austrian and Belgian pools were 0.411 and 0.375, respectively. Furthermore, the values of *PIC* of the Austrian and Belgian pools were 0.337 and 0.298, respectively.

**Figure 1:**
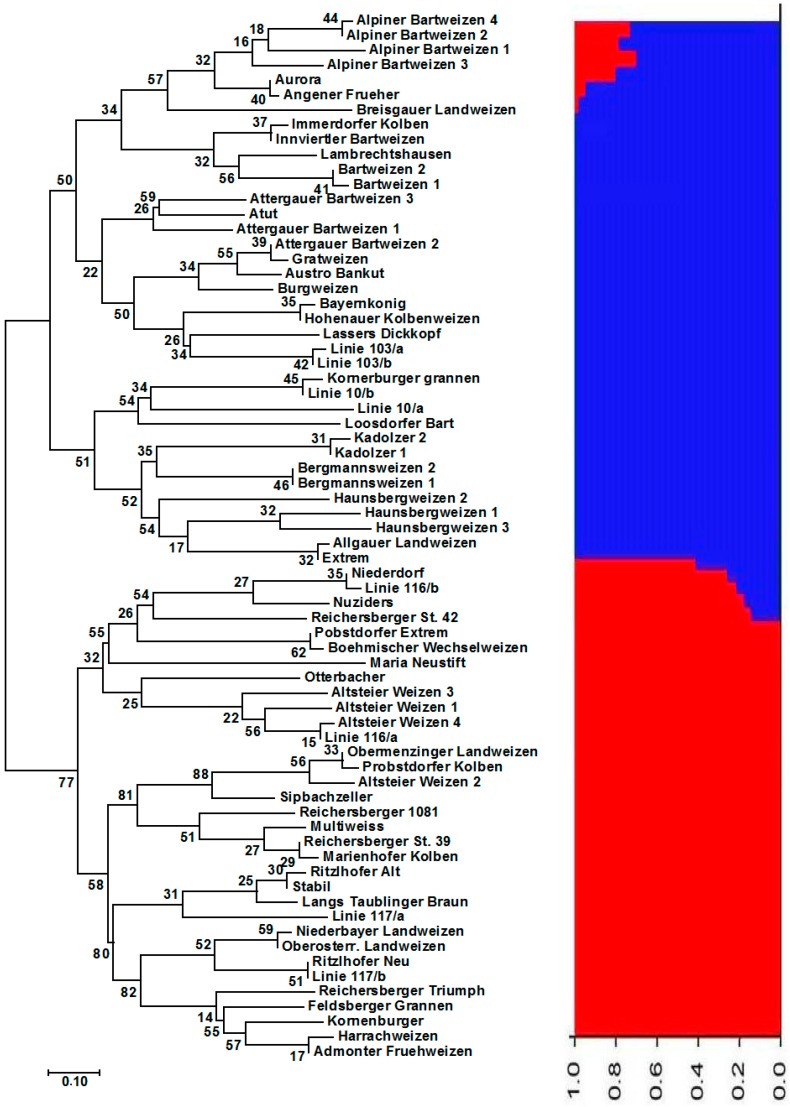
Neighbor-joining tree of the 70 Austrian wheat genotypes and their population structure (at *K* = 2) based on 1052 diversity array technology (DArT) markers. The numbers at the nodes of the phylogenetic tree represent the bootstrap values showing the probability of branching at 1000 replications. The red and blue colors represent two different populations.

**Figure 2:**
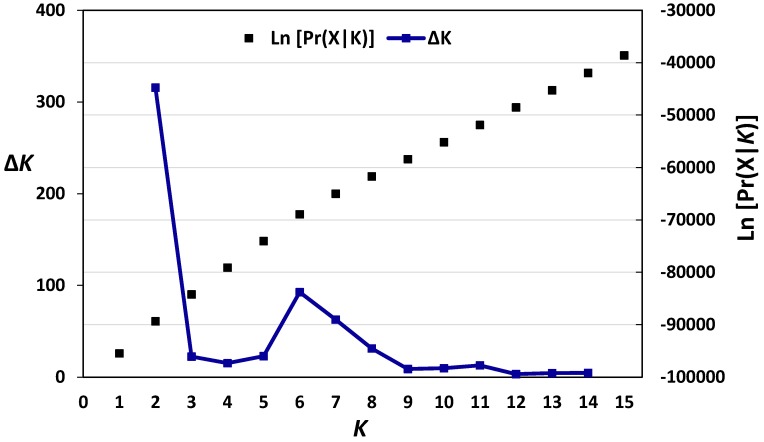
The log-likelihood and Δ*K* values based on the change rate of log-likelihood function between successive *K* [41] in the 70 Austrian wheat genotypes.

**Figure 3:**
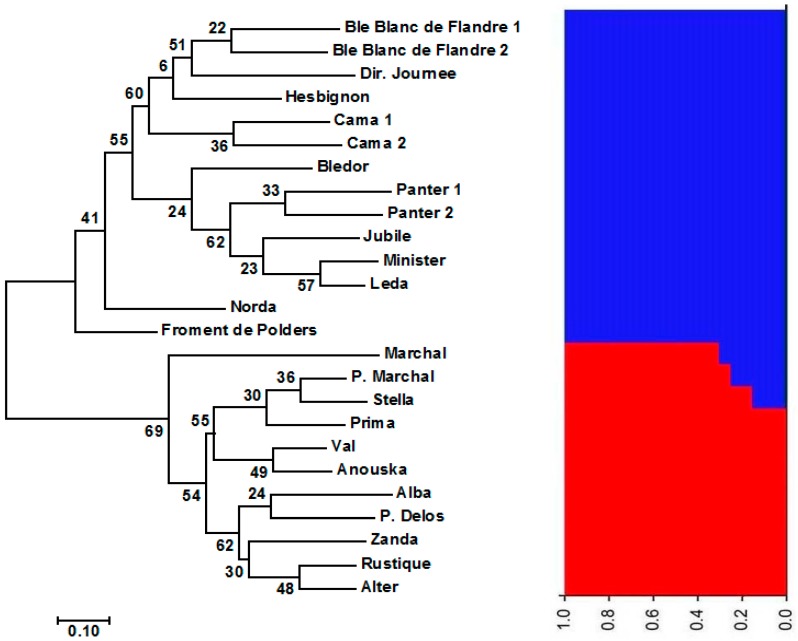
Neighbor-joining tree of the 25 Belgian wheat genotypes and their population structure (at *K* = 2) based on 1052 DArT markers. The numbers at the nodes of the phylogenetic tree represent the bootstrap values showing the probability of branching at 1000 replications. The red and blue colors represent two different populations.

**Figure 4:**
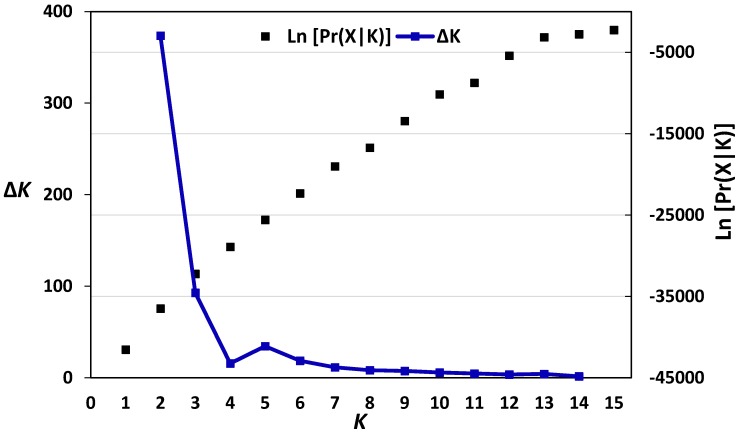
The log-likelihood and Δ*K* values based on the change rate of log-likelihood function between successive *K* [41] in the 25 Belgian wheat genotypes.

Jaccard dissimilarity matrix-based neighbour-joining tree performed for the Austrian wheat pool equally divided the Austrian pool into two main clusters (Figure 1). The first main cluster comprised 33 cultivars, whereas the second contained 37 genotypes. The structure analysis of the Austrian wheat genotypes showed that the highest Δ*K* value was recorded at *K* = 2 (315.68) followed by that at *K* = 6 (92.65) (Figure 2). At *K* = 2, the Austrian wheat genotypes were divided into two populations (A and B), in a complete concordance with the neighbour-joining tree results (Figure 1). Furthermore, the Jaccard dissimilarity matrix-based neighbour-joining tree performed for the Belgian wheat pool divided the Belgian pool into two main clusters (Figure 3). The first cluster contained 11 cultivars, whereas the second comprised 14 genotypes. The structure analysis of the Belgian wheat genotypes showed that the highest Δ*K* value was recorded at *K* = 2 (373.66) followed by that at *K* = 3 (92.78) (Figure 4). At *K* = 2, 11 Belgian wheat genotypes were assigned to population A, whereas population B comprised 14 genotypes in a complete concordance with the neighbour-joining tree results (Figure 3).

Analysis of molecular variance was conducted to partition the molecular diversity between and within the populations (*K =* 2), generated by Structure analysis, of each of the Austrian and Belgian wheat breeding pools (Table 3). The results showed that the majority of the diversity was attributed to differences among varieties within populations for the Austrian and Belgian breeding pools (80% and 81%, respectively) (Table 3).

### 3.2. Diversity, Population Structure and Relationships among European Wheat Varieties from Nine Countries

To place the Austrian and Belgian wheat breeding pools in a wider European context, a total of 141 common DArTs were used to calculate the genetic diversity indices of 618 European wheat varieties that originated from nine countries (Table 4). The *A*_10_ varied from 1.224 (Denmark) to 1.397 (Austria) (Table 4). Moreover, the *H_E_* ranged from 0.250 (Denmark) to 0.372 (Austria). The *PIC* varied from 0.202 (Denmark) to 0.301 (Austria). The average value of the effective *N_E_* varied from 1.332 (Denmark) to 1.588 (Austria) (Table 4). These results revealed that the Central European countries (Austria, Croatia and Hungary) contained higher level of diversity than that of the Northern and Western European countries (Belgium, Germany, Denmark, Sweden, the UK and France).

Analysis of molecular variance revealed that the majority of genetic variation was attributed to differences among varieties within countries (67.83%, Table 3). The results also revealed a highly significant difference between the two regions (Northern and Western Europe vs. Central Europe; *p* < 0.0001), which accounted for 26.53% of the total variance, whereas the difference recorded among countries within regions represented 5.64% (Table 3).

**Figure 5:**
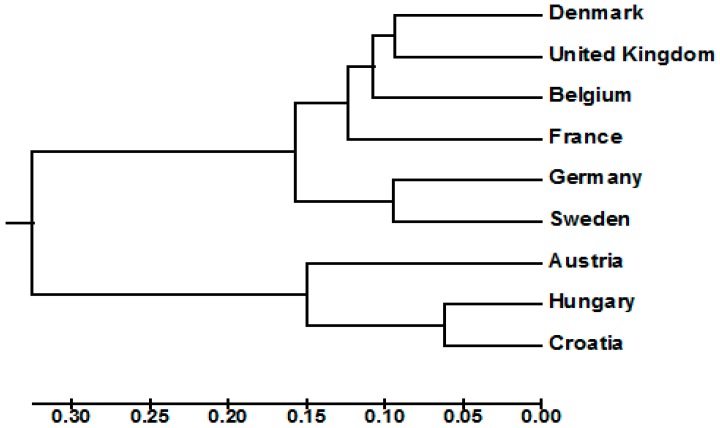
Unweighted pair group method with arithmetic mean (UPGMA) phylogenetic tree based on the genetic differentiation (*F_ST_*) values between countries.

UPGMA cluster analysis showed that the wheat genotypes of the Northern and Western European countries were clustered separately from the genotypes of the Central European countries (Austria, Croatia and Hungary) (Figure 5), indicating that the cultivars of the Central European countries comprised variation levels not currently represented in the Northern and Western European wheat breeding pool. Furthermore, the Northern and Western European wheat cluster was subdivided into two distinct subclusters. The first subcluster comprised the German and Swedish cultivars, while the second included the genotypes of Belgium, Denmark, the UK and France (Figure 5).

**Figure 6:**
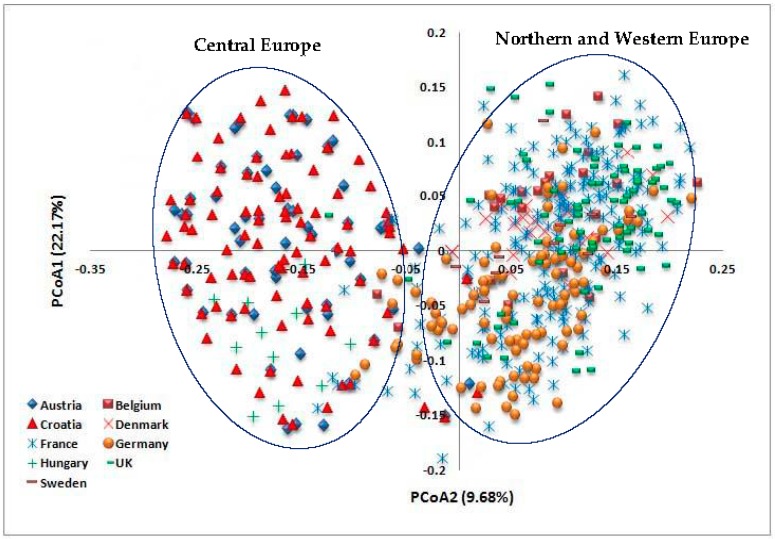
Principal coordinates analysis (PCoA) of 618 European wheat cultivars from nine countries.

Principal coordinate analysis also revealed the relationships among the 618 wheat cultivars from the nine European countries (Figure 6). The first two axes accounted for 22.17% and 9.68% of the total variance, respectively. The wheat genotypes from the Northern and Western European countries (Belgium, Denmark, Germany, Sweden, France and the UK) were clustered separately from the genotypes from the Central European countries (Austria, Croatia and Hungary) along the first axis.

**Figure 7:**
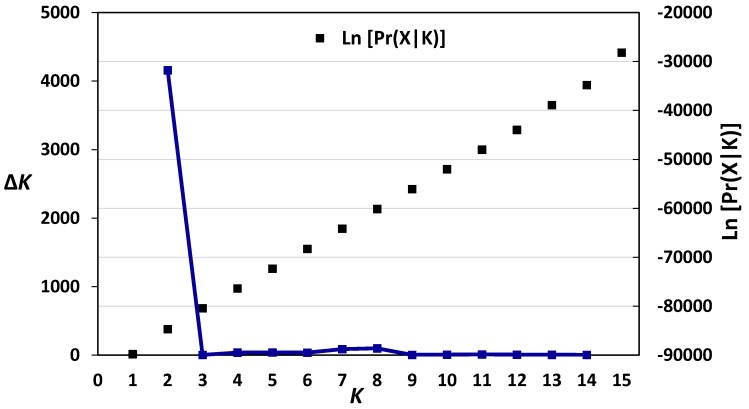
Analysis of population structure of 618 European wheat genotypes based on the 141 common DArT markers, the log-likelihood and Δ*K* values based on the change rate of log-likelihood function between successive *K* [41].

The structure analysis of the 618 European wheat genotypes showed that the highest Δ*K* value was recorded at *K* = 2 (4156.47) (Figure 7). Table 5 also shows the membership proportion of the 618 European wheat genotypes in each of the two populations (*K* = 2). The majority of wheat genotypes from the Northern and Western European countries were assigned to Population A, while the great majority of genotypes belonging to the Central European countries were assigned to population B (Table 5).

## 4. Discussion

Evaluation of the genetic diversity, population structure and relationships provide valuable information needed to broaden the narrow genetic base and to enhance breeding and conservation strategies of crops. DArT markers showed their efficiency in assessing the genetic diversity of different crops. Raman et al. [1] assessed the diversity of 1057 wheat genotypes collected from different regions worldwide using DArT markers that revealed an average PIC value of 0.44 and Nei’s diversity index of 0.43. Zhang et al. [38] also characterised 111 Chinese wheat genotypes using DArT markers that revealed an average value of 0.40 for both of PIC and Nei’s diversity index. Novoselović et al. [3] analysed 89 Croatian wheat genotypes using DArT markers and reported that the effective number of alleles per locus, PIC and expected heterozygosity were 1.64, 0.30 and 0.375, respectively. To the best of our knowledge, this is the first study to reveal the levels of genetic diversity and structure of the uncharacterised Austrian and Belgian wheat germplasm maintained at the CGN as well as place them in a wider European context. In this study, the characterization of Austrian and Belgian wheat pools showed different values for the diversity indices estimated. The rarefacted allelic richness recorded in the Austrian and Belgian breeding pools (*A*_25_ = 1.396 and 1.341, respectively) indicated that the Austrian breeding pool contained a slightly higher diversity than the Belgian pool. The neighbour-joining tree divided the Austrian germplasm into two main clusters, which are in a complete concordance with the structure analysis results. These results suggest that the breeders used different genetic material as parental resources, which resulted in genetically diverse cultivars. Furthermore, the neighbour-joining tree divided the Belgian germplasm into two main clusters. The first cluster contained 11 cultivars, whereas the second comprised the majority of cultivars, in a complete concordance with the structure analysis. This study also revealed that the majority of the diversity was attributed to differences among varieties within populations. These clustering and AMOVA results suggest that the crossing among inter-cluster cultivars may develop cultivars with promising agronomic traits.

Combining the DArT genotypes from three wheat panels previously published [3,34,35] with the Austrian and Belgian panels provides valuable assessment of diversity levels in a regional context. Rarefaction also facilitated the comparison of the diversity levels between the nine European pools. The *A*_10_ varied from 1.224 (Denmark) to 1.397 (Austria). The results revealed that the Central European countries (Austria, Croatia and Hungary) contained higher level of genetic diversity than that of the Northern and Western European countries (Belgium, Denmark, Germany, Sweden, France and the UK). This result is in accordance with that reported by Novoselović et al. [3] who found higher level of genetic variation in wheat genotypes originating from Central Europe, when compared to those from Northern and Western European countries. The higher level of diversity might be attributed to the higher number of alleles resulting from breeding and crossing practices [3,42,43].

Analysis of molecular variance revealed that the majority of genetic variation was attributed to the differences among varieties within countries (67.83%). This is in agreement with previous studies [3,43]. White et al. [44] also revealed the significant role of the geographical factor in genetic diversity studies. The results revealed a highly significant difference between the two geographical regions (Northern and Western Europe vs. Central Europe), which accounted for 26.53% of the total variance, suggesting that the cultivars of the Central European countries comprised variation levels not currently represented in the Northern and Western European wheat pool and could be used in future breeding and crop improvement programmes.

Unweighted pair group method with arithmetic mean cluster analysis showed that wheat genotypes of the Northern and Western European countries were clustered separately from the genotypes of the Central European countries. Within the Northern and Western European wheat cluster, the German and Swedish genotypes were separated in a distinct subcluster, in a complete accordance with the findings of Novoselović et al. [3]. Principal component analysis also revealed that the wheat genotypes from the Northern and Western European countries were clustered separately from the genotypes from the Central European countries, confirming our AMOVA results, which showed that the cultivars of the Central European countries comprised variation levels not currently represented in the Northern and Western European wheat breeding pool. The structure analysis divided the 618 European wheat genotypes into two populations. The majority of wheat genotypes from the Northern and Western European countries were assigned to one population, while the great majority of genotypes belonging to the Central European countries were assigned to the second population. These results are in accordance with our cluster analysis and PCoA data as well as with those previously reported by Novoselović et al. [3] and Le Couviour et al. [45].

In conclusion, the present study confirmed that the use of combined datasets, regional contribution made by plant breeders, and genotyping approaches could provide a promising opportunity for unlocking the genetic potential and improving conservation strategies of wheat. It is considered the first study that successfully revealed the high levels of genetic diversity and structure of the uncharacterised Austrian and Belgian wheat germplasm maintained at the CGN as well as facilitated placing them in a wider European context using efficient DArT markers. In addition, current results would help plant breeders to utilise and maintain the high levels of diversity recorded as well as selecting and using the most promising wheat genotypes in future breeding programs for enhancing and developing wheat cultivars of highly valuable agronomic traits.

## Figures and Tables

**Table 1:** Wheat breeding panels analysed in this study, including numbers of countries, cultivars and markers.

Wheat Panel	Number of Countries	Number of Cultivars	Markers	Number of Markers	Reference
Austrian Panel	1	70	DArT	1052	Current Study
Belgian Panel	1	25	DArT	1052	Current Study
TriticeaeGenome Panel	3	376	DArT/SNPs	2712/324	Bentley et al. [35]
European Diversity Panel	16	94	DArT	1849	Nielsen et al. [34]
Croatian Panel	1	89	DArT	1229	Novoselović et al. [3]
Combined dataset	9	618	DArT	141	

DArT, diversity array technology markers; SNPs, single nucleotide polymorphysms.

**Table 2:** Genetic diversity of the Austrian and Belgian wheat breeding panels based on 1052 DArT markers.

Wheat Panels	*N*	%*P*	*N_E_*	*A*_25_	*H_E_*	*PIC*
Austrian Panel	70	93.79	1.698	1.396	0.411	0.337
Belgian Panel	25	91.46	1.602	1.341	0.375	0.298

*N*, number of cultivars; %*P,* polymorphic markers percentage; *N_E_*, effective number of alleles; *A*_23_, rarefacted allelic richness (mean number of alleles rarefacted for a sample size of 25 cultivars); *H_E_*, expected heterozygosity; *PIC*, polymorphic information content.

**Table 3:** Analysis of molecular variance (AMOVA) of wheat panels from Austria (between and within populations), Belgium (between and within populations), and all nine European countries (between regions, among countries within regions, within regions).

Wheat Panels	Source of Variation	Variance Components	% Total Variance	*Probability*
Austrian Panel	Between populations	59.239	20%	0.0001
	Within populations	236.847	80%	
Belgian Panel	Between populations	40.161	19%	0.0001
	Within populations	169.985	81%	
All European Wheat Panels from Nine Countries *	Between regions	8.152	26.53%	0.0001
	Among countries Within regions	1.732	5.64%	0.0001
	Within countries	20.847	67.83%	0.0001

* Nine countries: Austria, Belgium, Croatia, Germany, France, Denmark, Sweden, Hungary and the UK.

**Table 4:** Genetic diversity of 618 European wheat varieties from nine countries estimated based on 141 DArT markers.

Country	*N*	%*P*	*N_E_*	*A*_10_	*H_E_*	*PIC*
Austria	70	92.8	1.588	1.397	0.372	0.301
Belgium	25	91.2	1.515	1.308	0.282	0.231
Croatia	89	100	1.538	1.331	0.351	0.296
Hungary	11	92.6	1.515	1.344	0.341	0.287
France	214	100	1.468	1.302	0.319	0.278
Germany	99	93.3	1.389	1.262	0.279	0.229
Denmark	22	90.7	1.332	1.224	0.250	0.202
Sweden	10	88.4	1.380	1.335	0.276	0.221
United Kingdom	78	90.5	1.333	1.248	0.251	0.204

*A*_10_, rarefacted allelic richness (mean number of alleles rarefacted for a sample size of 10 cultivars).

**Table 5:** Membership proportion of wheat varieties of each of the nine European counties in each of the two populations (*K* = 2) generated by Structure analysis [39].

Country	Membership in Population A (Pop. A) %	Membership in Population B (Pop. B) %
Austria	10	90
Croatia	25	75
Hungary	12	88
Germany	61	39
Sweden	67	33
France	79	21
Belgium	91	09
United Kingdom	87	13
Denmark	82	18

## Supplementary Materials

The following are available online at http://www.mdpi.com/2073-4425/9/1/47/s1. Table S1: List of Austrian and Belgian accessions analysed in this study.
