# Supplementary material for: Analysis of the Genetic Diversity and Population Structure of Austrian and Belgian Wheat Germplasm within a Regional Context Based on DArT Markers
Source: Genes (Basel). 2018 Jan 22;9(1):47. doi: 10.3390/genes9010047 (PMC5793198; doi:10.3390/genes9010047)
Supplement: Supplementary file 1 [file genes-09-00047-s001.pdf]

**Table S1. List of Austrian and Belgian accessions analyzed in this study.**

| No. | CGN Number | Accession name | Origin |
|---|---|---|---|
| 1 | CGN08888 | Admonter Fruehweizen | Austria |
| 2 | CGN14895 | Allgauer Landweizen | Austria |
| 3 | CGN14842 | Alpiner Bartweizen 1 | Austria |
| 4 | CGN14843 | Alpiner Bartweizen 2 | Austria |
| 5 | CGN14844 | Alpiner Bartweizen 3 | Austria |
| 6 | CGN14845 | Alpiner Bartweizen 4 | Austria |
| 7 | CGN14903 | Altsteier Weizen 1 | Austria |
| 8 | CGN14904 | Altsteier Weizen 2 | Austria |
| 9 | CGN14905 | Altsteier Weizen 3 | Austria |
| 10 | CGN14906 | Altsteier Weizen 4 | Austria |
| 11 | CGN08889 | Angener Frueher | Austria |
| 12 | CGN04281 | Attergauer Bartweizen 1 | Austria |
| 13 | CGN04282 | Attergauer Bartweizen 2 | Austria |
| 14 | CGN05364 | Attergauer Bartweizen 3 | Austria |
| 15 | CGN04479 | Atut | Austria |
| 16 | CGN09021 | Aurora | Austria |
| 17 | CGN08890 | Austro Bankut | Austria |
| 18 | CGN05374 | Bartweizen 1 | Austria |
| 19 | CGN05375 | Bartweizen 2 | Austria |
| 20 | CGN14780 | Bayernkonig | Austria |
| 21 | CGN04293 | Bergmannsweizen 1 | Austria |
| 22 | CGN05378 | Bergmannsweizen 2 | Austria |
| 23 | CGN08891 | Boehmischer Wechselweizen | Austria |
| 24 | CGN14900 | Breisgauer Landweizen | Austria |
| 25 | CGN12155 | Burgweizen | Austria |
| 26 | CGN08918 | Extrem | Austria |
| 27 | CGN05448 | Feldsberger Grannen | Austria |
| 28 | CGN05468 | Gratweizen | Austria |
| 29 | CGN08914 | Harrachweizen | Austria |
| 30 | CGN05481 | Haunsbergweizen 1 | Austria |
| 31 | CGN04355 | Haunsbergweizen 2 | Austria |
| 32 | CGN05482 | Haunsbergweizen 3 | Austria |
| 33 | CGN08892 | Hohenauer Kolbenweizen | Austria |
| 34 | CGN08893 | Immerdorfer Kolben | Austria |
| 35 | CGN04363 | Innviertler Bartweizen | Austria |
| 36 | CGN05507 | Kadolzer 1 | Austria |
| 37 | CGN05508 | Kadolzer 2 | Austria |
| 38 | CGN08897 | Kornenburger | Austria |
| 39 | CGN08896 | Kornerburger grannen | Austria |
| 40 | CGN14902 | Lambrechtshausen | Austria |
| 41 | CGN14894 | Langs Taublinger Braun | Austria |
| 42 | CGN08898 | Lassers Dickkopf | Austria |
| 43 | CGN14785 | Linie 10/a | Austria |
| 44 | CGN14786 | Linie 10/b | Austria |
| 45 | CGN14850 | Linie 103/a | Austria |
| 46 | CGN14851 | Linie 103/b | Austria |
| 47 | CGN14860 | Linie 116/a | Austria |
| 48 | CGN14861 | Linie 116/b | Austria |

| | | | |
|---|---|---|---|
| 49 | CGN14862 | Linie 117/a | Austria |
| 50 | CGN14863 | Linie 117/b | Austria |
| 51 | CGN08899 | Loosdorfer Bart | Austria |
| 52 | CGN04307 | Maria Neustift | Austria |
| 53 | CGN14787 | Marienhofer Kolben | Austria |
| 54 | CGN08919 | Multiweiss | Austria |
| 55 | CGN14893 | Niederbayer Landweizen | Austria |
| 56 | CGN04406 | Niederdorf | Austria |
| 57 | CGN05604 | Nuziders | Austria |
| 58 | CGN14897 | Obermenzinger Landweizen | Austria |
| 59 | CGN14899 | Oberosterr. Landweizen | Austria |
| 60 | CGN14846 | Otterbacher | Austria |
| 61 | CGN16137 | Pobstdorfer Extrem | Austria |
| 62 | CGN08904 | Probstdorfer Kolben | Austria |
| 63 | CGN08913 | Reichersberger 1081 | Austria |
| 64 | CGN08905 | Reichersberger St. 39 | Austria |
| 65 | CGN08906 | Reichersberger St. 42 | Austria |
| 66 | CGN08915 | Reichersberger Triumph | Austria |
| 67 | CGN04429 | Ritzlhofer Alt | Austria |
| 68 | CGN14901 | Ritzlhofer Neu | Austria |
| 69 | CGN05677 | Sipbachzeller | Austria |
| 70 | CGN08619 | Stabil | Austria |
| 71 | CGN08049 | Alba | Belgium |
| 72 | CGN08052 | Alter | Belgium |
| 73 | CGN08070 | Anouska | Belgium |
| 74 | CGN08059 | Ble Blanc de Flandre 1 | Belgium |
| 75 | CGN05387 | Ble Blanc de Flandre 2 | Belgium |
| 76 | CGN08060 | Bledor | Belgium |
| 77 | CGN12156 | Cama 1 | Belgium |
| 78 | CGN08069 | Cama 2 | Belgium |
| 79 | CGN08054 | Dir. Journee | Belgium |
| 80 | CGN05458 | Froment de Polders | Belgium |
| 81 | CGN08066 | Hesbignon | Belgium |
| 82 | CGN08050 | Jubile | Belgium |
| 83 | CGN08061 | Leda | Belgium |
| 84 | CGN12205 | Marchal | Belgium |
| 85 | CGN08062 | Minister | Belgium |
| 86 | CGN08068 | Norda | Belgium |
| 87 | CGN08063 | Panter 1 | Belgium |
| 88 | CGN16048 | Panter 2 | Belgium |
| 89 | CGN08055 | Prima | Belgium |
| 90 | CGN08053 | P. Delos | Belgium |
| 91 | CGN08067 | P. Marchal | Belgium |
| 92 | CGN08064 | Rustique | Belgium |
| 93 | CGN08065 | Stella | Belgium |
| 94 | CGN12254 | Val | Belgium |
| 95 | CGN08051 | Zanda | Belgium |
